# Are We Focused on the Wrong Early Postoperative Quality Metrics? Optimal Realignment Outweighs Perioperative Risk in Adult Spinal Deformity Surgery

**DOI:** 10.3390/jcm12175565

**Published:** 2023-08-26

**Authors:** Peter G. Passias, Tyler K. Williamson, Jamshaid M. Mir, Justin S. Smith, Virginie Lafage, Renaud Lafage, Breton Line, Alan H. Daniels, Jeffrey L. Gum, Andrew J. Schoenfeld, David Kojo Hamilton, Alex Soroceanu, Justin K. Scheer, Robert Eastlack, Gregory M. Mundis, Bassel Diebo, Khaled M. Kebaish, Richard A. Hostin, Munish C. Gupta, Han Jo Kim, Eric O. Klineberg, Christopher P. Ames, Robert A. Hart, Douglas C. Burton, Frank J. Schwab, Christopher I. Shaffrey, Shay Bess, on behalf of the International Spine Study Group

**Affiliations:** 1 Departments of Orthopaedic and Neurological Surgery, NYU Langone Orthopaedic Hospital, New York Spine Institute, New York, NY 10003, USA; 2 Department of Neurosurgery, University of Virginia, Charlottesville, VA 22904, USA; 3 Department of Orthopaedics, Lenox Hill Hospital, Northwell Health, New York, NY 10075, USA; 4 Department of Orthopaedics, Hospital for Special Surgery, New York, NY 10021, USA; 5 Department of Spine Surgery, Denver International Spine Clinic, Presbyterian St. Luke’s/Rocky Mountain Hospital for Children, Denver, CO 80205, USA; 6 Department of Orthopaedic Surgery, Warren Alpert School of Medicine, Brown University, Providence, RI 02912, USA; 7 Department of Orthopaedic Surgery, Norton Leatherman Spine Center, Louisville, KY 40202, USA; 8 Department of Orthopedic Surgery, Brigham and Women’s Center for Surgery and Public Health, Boston, MA 02120, USA; 9 Departments of Neurosurgery, University of Pittsburgh Medical Center, Pittsburgh, PA 15213, USA; 10 Department of Orthopaedic Surgery, University of Calgary, Calgary, AB T2N 1N4, Canada; 11 Department of Neurosurgery, University of California, San Francisco, CA 94143, USA; 12 Division of Orthopaedic Surgery, Scripps Clinic, La Jolla, CA 92037, USA; 13 Department of Orthopaedic Surgery, The Johns Hopkins Medical Institutions, Baltimore, MD 21205, USA; 14 Department of Orthopaedic Surgery, Baylor Scoliosis Center, Dallas, TX 75243, USA; 15 Department of Orthopedic Surgery, University of California Davis, Sacramento, CA 95819, USA; 16 Department of Orthopaedic Surgery, Swedish Neuroscience Institute, Seattle, WA 98122, USA; 17 Department of Orthopaedic Surgery, University of Kansas Medical Center, Kansas City, KS 66160, USA; 18 Spine Division, Departments of Neurosurgery and Orthopaedic Surgery, Duke University School of Medicine, Durham, NC 27710, USA; 19 Rocky Mountain Scoliosis and Spine, Denver, CO 80124, USA

**Keywords:** adult spinal deformity, cost-utility, complications, complex realignment, Medicare, clinical improvement

## Abstract

Background: While reimbursement is centered on 90-day outcomes, many patients may still achieve optimal, long-term outcomes following adult spinal deformity (ASD) surgery despite transient short-term complications. Objective: Compare long-term clinical success and cost-utility between patients achieving optimal realignment and suboptimally aligned peers. Study Design/Setting: Retrospective cohort study of a prospectively collected multicenter database. Methods: ASD patients with two-year (2Y) data included. Groups were propensity score matched (PSM) for age, frailty, body mass index (BMI), Charlson Comorbidity Index (CCI), and baseline deformity. Optimal radiographic criteria are defined as meeting low deformity in all three (Scoliosis Research Society) SRS-Schwab parameters or being proportioned in Global Alignment and Proportionality (GAP). Cost-per-QALY was calculated for each time point. Multivariable logistic regression analysis and ANCOVA (analysis of covariance) adjusting for baseline disability and deformity (pelvic incidence (PI), pelvic incidence minus lumbar lordosis (PI-LL)) were used to determine the significance of surgical details, complications, clinical outcomes, and cost-utility. Results: A total of 930 patients were considered. Following PSM, 253 “optimal” (O) and 253 “not optimal” (NO) patients were assessed. The O group underwent more invasive procedures and had more levels fused. Analysis of complications by two years showed that the O group suffered less overall major (38% vs. 52%, *p* = 0.021) and major mechanical complications (12% vs. 22%, *p* = 0.002), and less reoperations (23% vs. 33%, *p* = 0.008). Adjusted analysis revealed O patients more often met MCID (minimal clinically important difference) in SF-36 PCS, SRS-22 Pain, and Appearance. Cost-utility-adjusted analysis determined that the O group generated better cost-utility by one year and maintained lower overall cost and costs per QALY (both *p* < 0.001) at two years. Conclusions: Fewer late complications (mechanical and reoperations) are seen in optimally aligned patients, leading to better long-term cost-utility overall. Therefore, the current focus on avoiding short-term complications may be counterproductive, as achieving optimal surgical correction is critical for long-term success.

## 1. Introduction

Over the past twenty years, the landscape of adult spinal deformity (ASD) correction surgery has changed drastically in terms of the patient’s undergoing intervention and the strategies employed to optimize outcomes [1,2,3,4,5,6,7,8]. In particular, the last decade has witnessed the adoption of less invasive techniques to minimize complications and the identification of radiographic characteristics that can guide correction goals and lead to better long-term outcomes [9,10,11,12,13,14,15].

Given the invasiveness and intensity of these surgical interventions, the correction of spinal deformity does not provide instant satisfaction [16,17]. Previous work has consistently shown that the trajectory of recovery following ASD surgery is such that maximal improvement does not typically transpire until close to one-year postoperation [1,2]. Earlier studies examining factors influencing outcomes in this population saw no effect of complication occurrence on long-term clinical improvement [3,4]. Conversely, more recent studies with longer-term follow-up concluded that major complications later in the postoperative course, such as mechanical failure or proximal junctional kyphosis, played a detrimental role in the outcome of ASD surgery, often driving the difference between the best and worst outcomes [5,6]. It is also important to note that, despite the propensity for higher rates of complications in this surgical population, even patients at the highest risk for complications have been shown to achieve substantive clinical benefits [7,8,9,10,11,12].

Recently developed realignment strategies have sought to mitigate such late-occurring complications and maximize clinical improvement [13,14]. However, achieving such goals may necessitate substantive correction for those presenting with severe baseline deformity, demanding more invasive techniques and instrumentation. Surgeons must consider the trade-offs between the desire for optimal correction and the associated elevated risk of complications and extended recovery in the near term and the potential for long-term morbidity and increased healthcare utilization if the desired result is not achieved following index surgical intervention. This is also vital to understand considering the changing landscape of insurance surveillance of near-term outcomes and financial penalties that may follow from adverse events [15,18,19,20,21]. Our assumption is that the metrics currently used to assess the performance of surgery at 90 days are not reflective of the ultimate outcomes patients and surgeons hope to achieve.

Therefore, we sought to compare long-term clinical success and cost-utility between patients achieving optimal realignment and suboptimally aligned peers. Specifically, we focused on whether patients meeting optimal realignment endure similar rates of perioperative complications to those suboptimally aligned, and whether both groups go on to achieve long-term, durable outcomes, as well as comparable cost-utility following surgery. We hypothesized that optimal realignment would be associated with superior long-term outcomes and improved cost-utility, despite the elevated risk of complications in the immediate postoperative period.

## 2. Materials and Methods

### 2.1. Study Design and Inclusion Criteria

This study was a retrospective review of a prospective multicenter adult spinal deformity database. The means by which patients are eligible for inclusion, consented, followed, and had post-surgical data collected have been described in detail in previous works [16,22]. For this specific investigation, IRB approval was obtained to examine operative adult spinal deformity patients who had complete radiographic and health-related quality of life (HRQL) data at baseline and two-year follow-up.

### 2.2. Data Collection

We extracted demographic data for eligible individuals, including age, gender, body mass index (BMI), history of prior fusion, Passias-modified adult spinal deformity frailty index (modified ASD-FI), and baseline comorbidities categorized using the Charlson Comorbidity Index (CCI) [23,24]. Surgical parameters consisted of levels fused, operative time, length of stay, surgical approach, use of decompressions, and osteotomies. A standardized complication reporting form was completed for the perioperative time interval, for each clinical follow-up, and at any point, the site became aware of a new complication. Patient-reported outcome measures, prospectively collected at baseline and follow-up intervals, included the following: Short Form-36 (SF-36) questionnaire, Scoliosis Research Society Outcomes Questionnaire (SRS-22), pain Numerical Rating Scale for both leg (NRS-leg) and back (NRS-back), and the Oswestry Disability Index (ODI).

### 2.3. Radiographic Data Collection

Full length free-standing lateral spine radiographs (36-inch cassette) were collected and assessed at baseline and follow-up. Radiographic images were analyzed using SpineView^®^ (ENSAM, Laboratory of Biomechanics, Paris, France) software 2.0 according to standardized and validated techniques previously published in the literature [25,26,27].

### 2.4. Clinical Outcomes

To evaluate improvement in outcomes, minimum clinically important difference (MCID) thresholds were utilized based on published values in the literature for the SF-36 physical component score (PCS) and mental component score (MCS), ODI, and SRS-22 Pain, Mental, Activity, and Appearance [16,17,28,29,30,31]. ‘Best Clinical Outcome’ (BCO) by Smith et al. was defined as an ODI score less than 15 AND SRS-Total score greater than 4.5 by two years [6].

### 2.5. Complication Assessment

The reported complications were classified as minor or major, with complications that led to mortality, involved invasive intervention, or had prolonged or permanent morbidity, classified as major. Complications were grouped based on time of occurrence as perioperative (within 90 days of surgery, including hospital-acquired conditions (HACs; deep vein thrombosis (DVT), pulmonary embolism (PE), urinary tract infection (UTI), deep/superficial infection)) and longer-term (recorded from 90 days to at least two years following surgery) [32].

Radiographic criteria for proximal junctional kyphosis (PJK) and proximal junctional failure (PJF) were used, with PJK defined as a greater than 10° change from baseline in UIV +2 (upper instrumented vertebra) and greater than 10° angulation. PJF values were based on Lafage criteria of greater than 22° change from baseline in UIV to UIV +2 angulation along with the angle being greater than 28°.

### 2.6. Definition of Optimal Radiographic Criteria

Realignment goals for sagittal correction were analyzed. Previously published formulas for PI-LL (pelvic incidence minus lumbar lordosis), PT (pelvic tilt), and SVA (sagittal vertical axis), established by Schwab et al., were used [33]. Patients were considered low (0) deformity with the following parameters: PI-LL below 10°, PT below 20°, and SVA below 40 mm.

A Global Alignment and Proportion (GAP) score was generated for all included patients, consisting of five parameters, namely relative pelvic version (measured minus ideal sacral slope) [0–3], relative lumbar lordosis (measured minus ideal lumbar lordosis) [0–3], lordosis distribution index (L4-S1 lordosis divided by L1-S1 lordosis multiplied by 100) [0–3], and relative spinopelvic alignment (measured minus ideal global tilt) [0–3], as well as an age factor [0–1] [14].

Patients were categorized based on being corrected to ‘optimal radiographic criteria (O)’, defined as: meeting low deformity in all three SRS-Schwab parameters or being proportioned in GAP score postoperatively.

### 2.7. Propensity Score Matching

Using previously published methods, patients were propensity score matched for age, frailty, BMI, CCI, and baseline deformity (PI-LL, PT, and SVA).

### 2.8. Cost Calculation

In line with prior work, the PearlDiver database was utilized to calculate costs using job order cost accounting (“charge analysis”) [22]. Reflecting both Medicare reimbursement and private insurance, PearlDiver data are some of the most comprehensive datasets with access to Medicare reimbursement charges, outcome data, and trends. Using mean costs associated with procedures based on 2018 adult spinal deformity diagnosis-related groups, procedural costs for cases, cases with complications and comorbidities (CC), major complications and comorbidities (MCC), and revisions were determined according to CMS.gov manual definitions [34]. Our estimates for two-year reimbursement consisted of a standardized determination using regression analysis of Medicare pay-scales for all services rendered within a 30-day window, including costs of postoperative complications, outpatient healthcare encounters, revisions, and medical-related readmissions, as per previously published methods [35,36,37]. The World Health Organization (WHO) has determined the threshold value for cost-effectiveness when analyzing cost-utility ratios; therefore, $187,818 represents the upper threshold of the United States cost/QALY (quality-adjusted life years) willingness to pay, and values above this were deemed ‘Low Cost-Utility’ [25,37,38,39,40].

### 2.9. Utility Calculation

Utility data were calculated via the difference between the baseline and the corresponding ODI score at the follow-up time point (six weeks, one year, and two years). Utility data were calculated using ODI converted to SF-6D and subsequently to QALYs using published conversion methods [40,41].

### 2.10. Statistical Analysis

The primary outcome was cost per QALY at two years. Secondary outcomes included the following: complication rates and clinical HRQL outcomes. Baseline data were compared between the cohorts using chi-squared and *t*-tests. Multivariate analysis controlling for baseline demographics was used to determine significant associations between achieving optimal radiographic criteria and each outcome variable. Analysis of covariance (ANCOVA) controlling for baseline disability and deformity (PI, PI-LL) were used to determine the utility gained and cost per QALY at six weeks, one year, and two years stratified by meeting optimal radiographic criteria. All *p*-values less than 0.05 were considered significant. All statistical analyses were conducted using IBM SPSS (Statistical Package for Social Sciences), version 25.0 (Armonk, NY, USA).

## 3. Result

### 3.1. Patient Demographics

**Figure 1:**
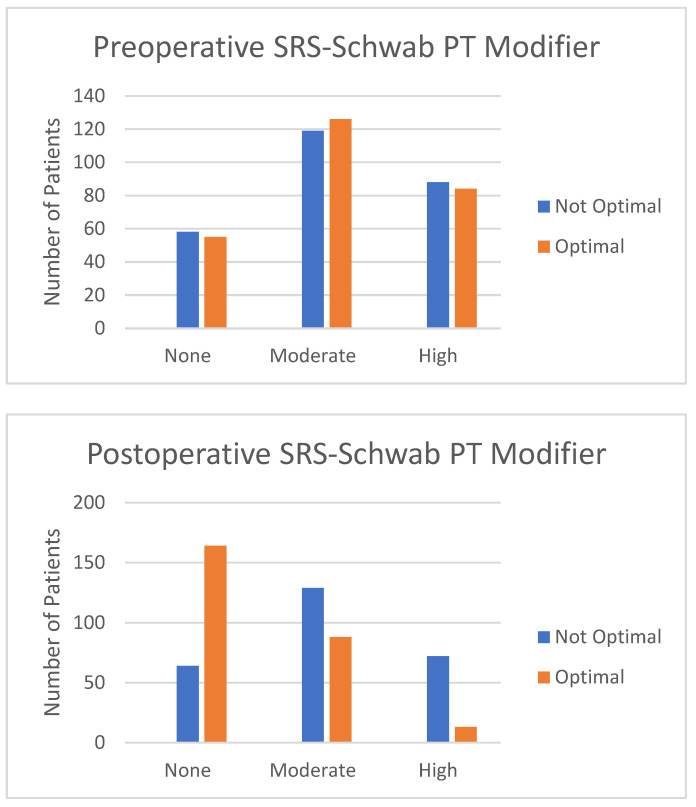

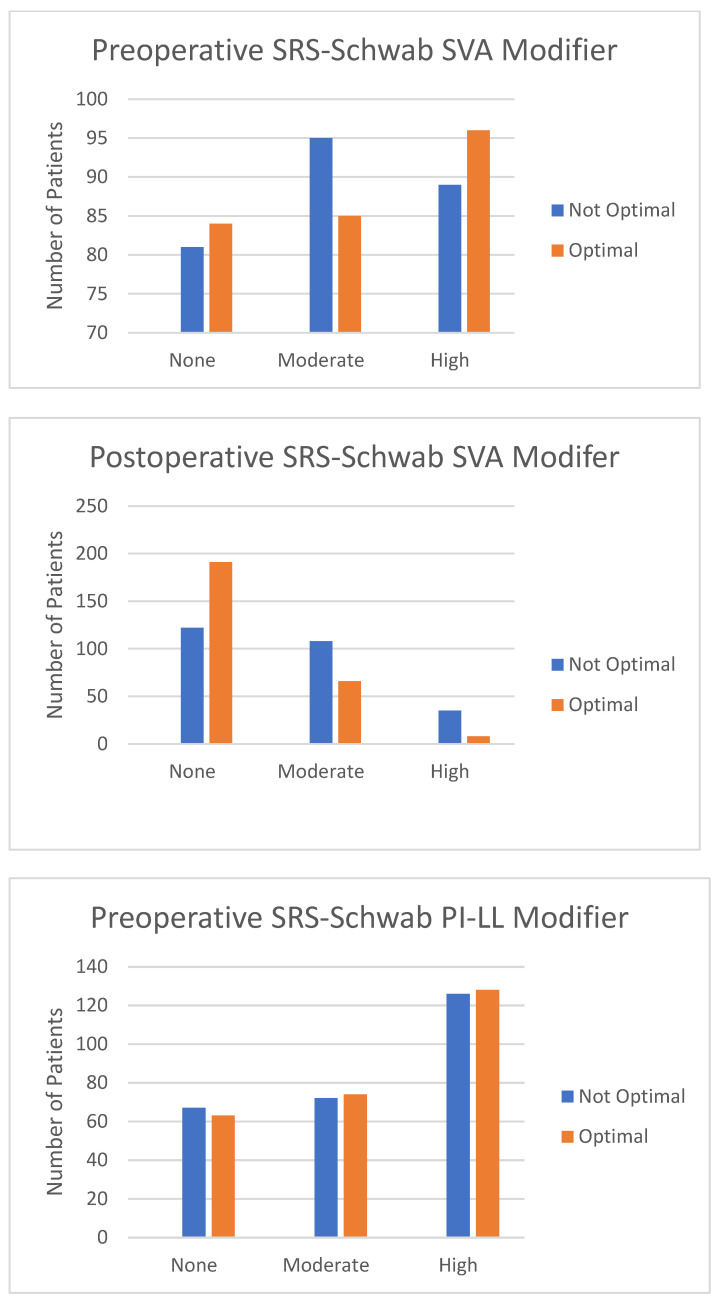

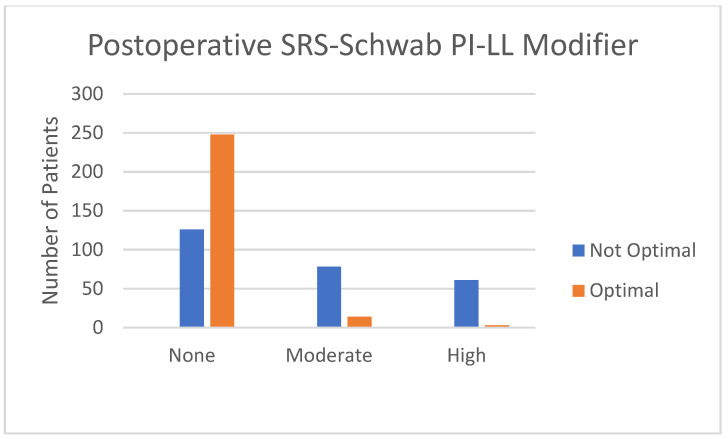
SRS-Schwab modifiers preoperatively and postoperatively.

**Figure 2:**
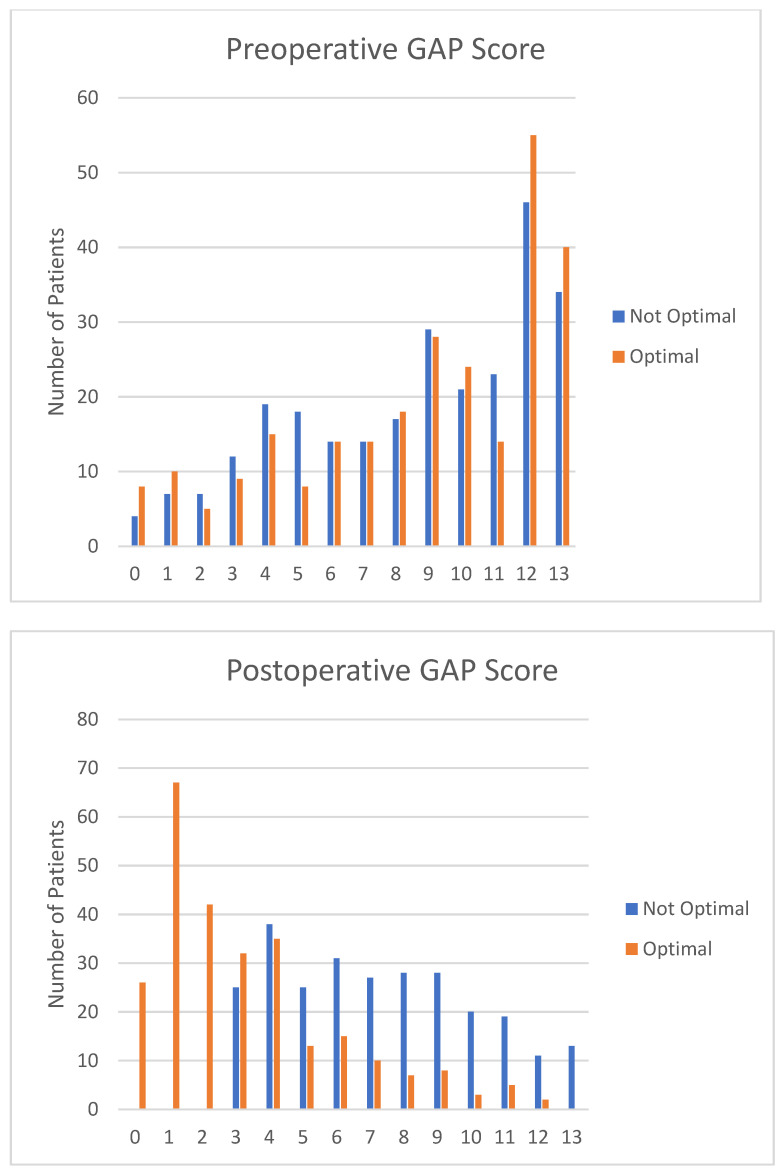

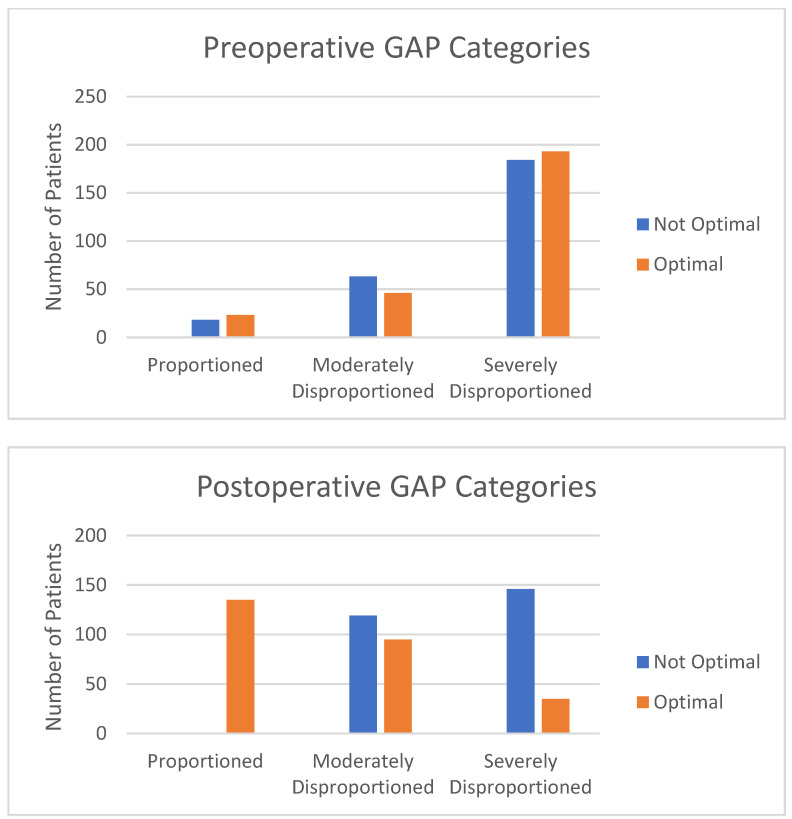
GAP score and proportionality preopertively and postoperatively.

Of the total cohort, 930 patients met radiographic and clinical follow-up criteria (mean patient age: 60.2 ± 14.3 years, BMI: 27.9 ± 5.8 kg/m^2^, Charlson Comorbidity Index (CCI): 1.8 ± 1.7, modified ASD Frailty Index: 7.5 ± 4.9, 76% female). The correction resulted in 40.1% of patients meeting low deformity for all three SRS-Schwab parameters and 34% being proportioned in GAP. There were 106 patients (11.4% of the cohort) proportioned in GAP and meeting all SRS-Schwab criteria. Figure 1 and Figure 2 depict preoperative and postoperative SRS-Schwab and GAP scores.

### 3.2. Categorization

The patient demographics categorized 467 patients (50.2%) into meeting optimal radiographic criteria and 463 (49.8%) were “not optimal” (NO). Regarding baseline demographic and radiographic assessment, groups were different in age, BMI, CCI, frailty, as well as SVA, PI-LL, and PT (all *p* < 0.05). Groups were propensity score matched for these significant confounders, generating 253 in O group and 253 in NO group.

### 3.3. Comparison of Surgical Details and Hospital Stay Based on Radiographic Outcomes

The O group endured similar operative time and EBL (both *p* > 0.2), but had a significantly higher ASD-SR invasiveness score (104 vs. 83, *p* < 0.001) with more overall osteotomies (83% vs. 65%, *p* < 0.001), three-column osteotomies (3COs) (25% vs. 15%, *p* = 0.006), and levels fused (11.8 vs. 10.1, *p* < 0.001; Table 1). The O group more often incorporated the use of multiple posterior support rod constructs compared to the NO group (Odds ratio (OR): 1.8, [1.1–2.8]; *p* = 0.010) and fixation to the pelvis (OR: 1.9, [1.04–2.6]; *p* = 0.036).

### 3.4. Comparison of Early Metrics during 90-Day Perioperative Course

Examining hospital course, groups had similar rates of intraoperative and in-hospital complications, surgical intensive care unit (SICU) admissions, and time, leading to an equivocal length of stay (all *p* > 0.2). Analysis of perioperative complications showed that the O group suffered similar rates of perioperative complications (50.0% vs. 49.4% in the NO group; *p* = 0.862) and rates of hospital-acquired conditions (HACs) (9.4% vs. 9.8%, *p* = 0.883; Table 2).

### 3.5. Complication Rates by Two Years

Analysis of complication rates by two years showed that patients in the O group suffered lower rates of overall major complications (38.4% vs. 51.9%, *p* = 0.021), major mechanical complications (12.1% vs. 22.4%, *p* = 0.002), mechanical complications requiring reoperations (8.0% vs. 18.2%, *p* = 0.001), and overall reoperations (22.6% vs. 32.5%; *p* = 0.008; Table 2).

Contributors to the rates of major mechanical complications were in NO compared to O groups: implant dislocation (3% vs. 0%, *p* = 0.033), rod breakage (15% vs. 11%, *p* = 0.166), screw breakage (4% vs. 2%, *p* = 0.104), screw loose (2% vs. 0%, *p* = 0.006), and pseudarthrosis (6% vs. 3%, *p* = 0.088).

Despite mechanical complication rates being lower in O, rates of PJK and PJF were higher, with PJK rates being slightly higher in O (44.9% vs. 37.0%, *p* = 0.064); this difference was not statistically significant. Radiographic PJF rates were higher in O (12.8% vs. 7.6%, *p* = 0.044).

### 3.6. Patient-Reported Outcomes by Two Years

Adjusted analysis revealed that patients in the O group more often met MCID in SF-36 PCS (64% vs. 45%, *p* = 0.001), SRS-22 Appearance (74% vs. 57%, *p* = 0.001), and SRS-22 Pain (71% vs. 60%, *p* = 0.042) by two years. When controlling for baseline deformity, age, BMI, frailty, and the use of 3CO, optimal outcome patients more often met the best clinical outcome (OR: 1.8, [1.1–3.2]; *p* = 0.027; Table 3).

### 3.7. Cost Analysis

When examining upfront cost accounting for the surgical approach, considering complications within 90 days, analysis determined no difference between the two groups in cost, as seen in Table 4 (O: $62,406.37 vs. NO: $63,623.07, *p* = 0.4). However, when accounting for the cost accumulated from complications and reoperations for up to two years, the O group demonstrated significantly lower cost ($93,727.98 vs. $108,320.30, *p* = 0.002; Table 4).

### 3.8. Cost-Utility Trend Analysis

The adjusted analysis determined no difference between the two groups in utility gained or cost-utility by six weeks, as depicted in Table 4. The O group generated better cost-utility by one year ($196,186.90 vs. $234,966.79, *p* < 0.001), and expanded by two years ($97,553.18 vs. $117,488.03, *p* < 0.001). When examining patients above the two-year WHO willingness-to-pay threshold of $187,818, there was a greater proportion of patients in the NO group above this threshold compared to the O group (32.3% vs. 21.1%, *p* = 0.004).

## 4. Discussion

Surgical correction for adult spinal deformity has proven to provide value for patients, despite a heightened morbidity profile and associated costs of care [42,43,44,45]. Prior to this work, a question remained whether performing less intensive procedures that resulted in suboptimal correction might be preferable to achieving idealized correction if it meant avoiding near-term complications and delayed recovery. However, our study indicates that, despite similar perioperative complication rates to those with suboptimal realignment, those achieving optimal realignment targets demonstrated superior clinical improvement, lower rates of reoperation, and ultimately better cost-utility at both one and two years.

Throughout the previous literature, there are mixed recommendations on the use of realignment targets based on which realignment classification schematics are superior in producing better clinical outcomes and reducing complications. Therefore, we chose criteria inclusive of two different classifications to reflect the clinical reality that a surgeon may use different realignment classifications, but adequate correction to target remains the most important goal of ASD surgery. We found that nearly half of our cohort achieved optimal realignment criteria. As this group differs significantly at presentation, we used propensity score matching to balance cohorts based on attempted correction during surgery.

Within the matched cohorts, we did not observe significant differences in the occurrence of complications during the 90-day period following surgery. These complications have been shown to have little impact on postoperative clinical improvement at final follow-up in previous studies [11,12]. However, healthcare costs associated with the management of near-term complications pale in comparison to the staggering economic impact of mechanical complications and late-term junctional kyphosis, which often doubles the cost of care [46,47,48]. By two years, we witnessed considerable rates of mechanical complications (e.g., implant failure), radiographic complications, and reoperations, yet lower such rates in optimally aligned patients.

By two years, we also observed that optimally aligned patients showed superior improvements in SF-36 PCS and SRS-22 Pain and Appearance, as well as meeting BCO more often. While minimizing complications is important to prevent disruptions in improvement during the postoperative course, restoring quality and functionality within everyday activities remains the ultimate goal for patients undergoing ASD surgery.

The effect of surgical approach, patient-specific factors, and certain interventions has been examined for their effect on cost-utility in the realm of ASD correction, with previous studies linking radiographic data to cost-utility having only focused on baseline measurements, showing a higher severity of deformity was a preoperative predictor of poor cost-utility [48,49,50]. However, these investigations did not account for the extent of surgical correction when analyzing cost-utility. This is the first study we are aware of to demonstrate the clinical and economic utility of achieving optimal alignment while accounting for baseline deformity in an ASD cohort.

The combination of clinical improvement that resulted in increased utility gained and lower rates of complications culminated in a lower cost-utility for optimally aligned patients at two years. Interestingly, significant differences in cost-utility were not encountered at six weeks, but rather only began to manifest at the one-year time point. Yet, reimbursement for services in adult spinal deformity is assessed by the outcomes within 90 days, much earlier than the time point where distinctions can be made regarding favorable long-term outcomes. This is an important fact to recognize on the part of surgeons, hospitals, and third-party payers, and suggests further lines of research along ways to better align payment models with outcomes when the recovery process exists on the type of delayed timeline evident for ASD surgery.

We do acknowledge several inherent limitations. First, this remains a retrospective work with the potential for selection, indication, surveillance, and classification bias. We attempted to adjust for this to the fullest extent possible using propensity score matching techniques but recognize the prospect for residual confounding to impact our determinations. Second, we utilized Medicare-allowable rates for our cost comparison. We felt Medicare rates would represent a suitable means of standardizing costs across different participating centers and improve the generalizability of study findings, although this may limit translational capacity to other payors [35]. Although this study included patients that had a minimum of two-year follow-up, which captures the majority of complications that may arise, it does not assess longer-term complications that may arise which is a limitation of this study. Furthermore, a limitation of SRS-Schwab is that it does not factor in overcorrection and is therefore a limitation of this study for patients that were included in the optimal alignment cohort based on SRS-Schwab solely. We surmise the higher PJK rates in the optimal alignment cohort to be attributed to those that were overcorrected and met SRS-Schwab, rather than meeting GAP proportionality. Future studies should assess the durability of realignment strategies in long-term studies to further delineate the attributable benefit of meeting radiographic targets.

## 5. Conclusions

Surgical intervention for adult deformity is associated with a heightened risk of postoperative complications, irrespective of final radiographic alignment. Fewer late complications (mechanical and reoperations) are seen in optimally aligned patients, leading to better long-term cost-utility overall. Therefore, the current focus on avoiding short-term complications may be counterproductive, as achieving optimal surgical correction is critical for long-term success.

## Figures and Tables

**Table 1:** Surgical differences between meeting optimal radiographic criteria.

	Not Optimal	Optimal	*p*-Value
Surgical/Admission Characteristic			
Number of Levels Fused	10.1 ± 4.6 levels	11.8 ± 3.9 levels	<0.001
Estimated Blood Loss	1684 ± 1588 mL	1829 ± 1386 mL	0.264
Operative Time	448 ± 188 min	465 ± 190 min	0.299
Osteotomy	65%	83%	<0.001
3CO	15%	25%	0.006
Decompression	61%	68%	0.065
Invasiveness Index	83.3	104.3	<0.001
Length of Stay	7.8 ± 4.3 days	8.3 ± 6.2 days	0.231
SICU Admission	67%	71%	0.303

**Table 2:** Group differences in perioperative and long-term complications.

	Not Optimal	Optimal	*p*-Value
Complications within 90 Days			
Adverse Event	12.1%	16.6%	0.137
Hospital-Acquired Condition	9.8%	9.4%	0.883
Any Perioperative Complication	49.4%	50.2%	0.862
Medical	7.9%	10.6%	0.295
Neurological	3.4%	1.1%	0.080
Pulmonary	4.2%	4.5%	0.832
Renal	1.1%	0.4%	0.316
Musculoskeletal	3.4%	3.8%	0.816
Cardiac	0.8%	1.5%	0.413
Gastrointestinal	5.3%	5.3%	0.999
Infection	5.7%	4.2%	0.422
Two-Year Complications			
Any Complication	72.1%	70.6%	0.702
Major Complication	51.9%	38.4%	0.021
Minor Complication	40.0%	36.6%	0.422
Mechanical Complication	44.2%	31.7%	0.003
Major Mechanical Complications	22.4%	12.1%	0.002
Mechanical Complications Requiring Reoperation	18.2%	8.0%	0.001
Reoperation	32.5%	22.6%	0.008

**Table 3:** Group differences in 2 Y patient-reported outcomes.

Outcome	Not Optimal	Optimal	*p*-Value
MCID in ODI	47%	53%	0.218
MCID in SRS-22 Pain	60%	71%	0.042
MCID in SRS-22 Appearance	57%	74%	0.001
MCID in SRS-22 Activity	56%	60%	0.531
MCID in SF-36 PCS	45%	64%	0.001
Best Clinical Outcome	8.8%	15.1%	0.025

**Table 4:** Cost-utility trend analysis of meeting optimal radiographic outcome.

	Did Not Meet Optimal Radiographic Outcome	Met Optimal Radiographic Outcome	*p*-Value
6 Weeks			
Utility Gained	0.552	0.548	0.837
Week 6 QALYs	0.063	0.063	0.837
Upfront Cost	$63,623.07	$62,406.37	0.399
Week 6 Cost per QALY	$1,138,113.98	$1,081,398.68	0.261
1 Year			
Utility Gained	0.468	0.485	0.184
Year 1 QALYs	0.461	0.477	0.184
Year 1 Cost per QALY	$234,966.79	$196,186.90	<0.001
2 Year			
Utility Gained	0.475	0.495	0.108
Year 2 QALYs	0.922	0.961	0.108
Year 2 Overall Cost	$108,320.30	$93,727.98	0.002
Year 2 Cost per QALY	$117,488.03	$97,553.18	<0.001

## Data Availability

The data presented in this study are available on request from the corresponding author. The data are not publicly available due to patient health data.
